# Male partner involvement in efforts to eliminate mother-to-child transmission of HIV in Kisumu County, Western Kenya, 2015

**DOI:** 10.11604/pamj.supp.2017.28.1.9283

**Published:** 2017-11-04

**Authors:** Elvis Oyugi, Zeinab Gura, Waqo Boru, Jane Githuku, Dickens Onyango, Walter Otieno, Venny Nyambati

**Affiliations:** 1 Jomo Kenyatta University of Agriculture and Technology, Kenya; 2 Field Epidemiology and Laboratory Training Program, Ministry of Health, Kenya; 3 County Health Department, Kisumu County, Kenya; 4 Maseno University, Kenya

**Keywords:** Male involvement, HIV transmission, elimination, Kisumu, Kenya

## Abstract

**Introduction:**

male partner involvement in elimination of mother-to-child transmission (eMTCT) of HIV activities remains low in Western Kenya, despite its importance in reducing rates of child HIV transmission. We sought to identify factors associated with male partner involvement in eMTCT in Kisumu East sub-County, Western Kenya.

**Methods:**

we conducted a cross-sectional study among women aged ≥ 18 years who had children aged ≤ 12 months and were attending a child health clinic for immunization services in one of four Western Kenya health centers between February and April, 2015. We assessed male involvement using an "involvement index" of five factors of equal weight: partner antenatal care (ANC) attendance, partner HIV testing, partner financial support to the woman during ANC, partner awareness of ANC services and partner participation in decision making on contraception including condom use. Male involvement was classified as high or low based on their index score. We calculated odds ratios (OR) and 95% confidence intervals (CI) to identify factors associated with high male partner involvement.

**Results:**

we recruited 216 female participants. Mean age was 26.1 years (± 5.5 years), 189 (87.5%) were married. The majority (94.4%) had attended ANC in public health facilities. Nineteen percent of women had high male involvement. Having > 8 years of formal education (AOR 3.9, CI = 1.51-10.08), having male partner who was employed, history of previous couple testing (AOR = 3.2, CI = 1.42-7.22) and reports of partner having read the mother-child booklet during ANC (AOR = 2.9, CI = 1.30-6.49), were associated with high male involvement.

**Conclusion:**

based on our findings, we recommend targeted strategies to actively sensitize men and encourage their involvement in eMTCT, particularly among partners of women with fewer years of education and among partners who are not employed.

## Introduction

The transmission of human immunodeficiency virus (HIV) from an infected woman to her child during pregnancy, labor, delivery or breastfeeding periods is referred to as mother-to-child transmission (MTCT) [1]. Without any interventions during these stages, MTCT rates can range from 15% to 45%, but these rates can be reduced to levels below 5% with effective interventions [2]. Male partner involvement is a key strategy in efforts to eliminate MTCT and has been shown to reduce rates of MTCT by as high as 40% [1,3,4]. Male partner involvement can also aid in the identification of sero-discordant couples at the ANC and improve anti-retroviral therapy (ART) adherence by HIV-infected women, which subsequently increases likelihood of having HIV-exposed children who test negative at18 months of age [5,6]. Kenya currently has a HIV prevalence of 5.6% among adults [7] and the national average MTCT rate between 2007-2012 was 15% [8], which was higher than the desired levels of 5% or below [2]. Low male partner participation in activities geared towards elimination of MTCT (eMTCT) has been cited as one of the reasons for the high rate of MTCT in Kenya [9,10].

In 2011, the United Nations joint agency for HIV and AIDS (UNAIDS) launched an initiative that aimed at eliminating MTCT to levels below 5% by the year 2015. The initiative sought to enable all pregnant women access to HIV testing and care services and to prevent new HIV infections among women of reproductive age [10]. It prioritized the sub Saharan Africa region because the region accounted for up to 90% of new HIV infections among children globally [11] and continues to have low male involvement in female reproductive health activities, including antenatal care (ANC) and eMTCT services [5,12-15]. Though male involvement is considered important in eMTCT, it currently has no standard definition. Some consider that partner HIV testing at the ANC is considered to be male involvement [5,12,16-18], while other studies have created indices to quantify male involvement. Male partner awareness of woman’s ANC appointments, visit to the ANC, awareness of ANC interventions, acceptance of HIV testing at the ANC, and financial support to the woman for ANC attendance are contributors that have been in male involvement indices in the past [16,17,19].

In Kenya, past studies that assessed involvement based on male partner testing for HIV at the ANC reported that male involvement ranged between 10-16.5% among women obtaining services [6,20-22]. One of the reasons attributed to this low proportion is that men prefer HIV testing at alternative sites rather than the ANC, such as voluntary testing and counseling centers or home-based testing [23]. Although Western Kenya has the highest burden of HIV infection in the country, male involvement in the region is thought to be low, and there have been no published data on the factors that influence the involvement of men in eMTCT services in the Western Kenya region. Therefore, we sought to estimate the proportion of women with male involvement in eMTCT activities and to describe the factors associated with male involvement in Kisumu East sub County.

## Methods

### Study settings

The study was conducted in Kisumu East sub-County in Kisumu County of Western Kenya region. The sub-County consists of two administrative divisions: Winam, which is predominantly urban, and Kadibo, which is rural. Kisumu East sub-County is predominantly inhabited by the Luo community. In 2013, 24% of the population were women of childbearing age (15-49 years) and 3.8% were pregnant women [24]. The HIV prevalence in Western Kenya is 15.1% among adults [7]. There were 50 health facilities within the sub-County offering basic health services including HIV care, with a total of four health centers, two located in each division.

### Study design and population

We conducted a facility-based cross-sectional study between February and April 2015. The study population was women aged ≥ 18 years who had delivered a child ≤ 12 months prior to the study period, and were attending child immunization services at one of the four health centers during the study period.

### Sample size determination

We used Cochran’s formula [25] to calculate the sample size required to estimate proportion of women with male involvement in eMTCT activities in the study area assuming 15% male partner involvement in eMTCT [6] and 5% precision. The minimum sample size was 216 participants after adjusting for 10% non-response.

### Sampling procedures

The study area has four heath centers from which women were selected for the study. Systematic random sampling method was used to select study participants. Based on information regarding the first ANC visit workload for the health centers during 2013, from the Kenya Health Information System [24], we applied probability-proportional-to-size sampling to determine the sample size for each health center. Our sampling interval was based on the daily entries in the mother-child health (MCH) register in January 2015. A table of random numbers was used to select the first participant for interview, and we subsequently sought to enroll every fifth entry in the MCH register for interview following informed and voluntary consent. If a randomly-selected participant was not eligible for interview or refused to be part of the study, the next eligible participant on the list was selected. Only one visit for child immunization was considered for enrollment, and already enrolled participants were no longer eligible to be selected in case they re-visited the clinic.

### Eligibility criteria

To be eligible for enrollment, the woman was required to be at least 18 years of age and to have made at least one ANC visit to a health facility within the subcounty during pregnancy. She must also have had a male partner during pregnancy, regardless of her marital status.

### Male partner involvement index

We adapted an index used to assess male involvement by Byamugisha et al. in a study in Uganda [16] which consisted of the following contributors: 1) The woman was accompanied by the man to the ANC on at least one visit; 2) The man was tested for HIV during any one of the ANC visits; 3) The woman reported that the man was aware of the woman’s ANC appointments and discussed ANC interventions with the woman; 4) The man consulted the woman on the use of condoms and other contraception; 5) The man provided financial support for the woman’s ANC attendance on at least one occasion; 6) Each contributor was given an equal weight in the index. An involvement score ranged from 0-5. The cut off for high male involvement was set at half of the index as has been done in past studies [16,17,19]. A total score of 0-2 was considered low male involvement while a score of 3-5 was considered as high male involvement.

### Data collection

We interviewed women using a pre-tested standardized questionnaire to collect information on their socio-demographic characteristics, their male partners’ characteristics, and the factors associated with male involvement. The questionnaire was translated into Luo and Swahili languages and back-translated to English for validation of accuracy. We reviewed the mother-child booklet to verify the mother’s socio demographic status and ANC profiles. The outcome of interest for bivariate analysis was high male involvement using the male partner involvement index compared to low male involvement. We assessed the outcome and its relationship to sociodemographic characteristics of the participants (age, marital status, religion, residence, employment status, level of education, number of children, and type of marriage), and male partner awareness of ANC activities (read mother-child booklet).

### Data analysis

Data was entered, cleaned and analyzed using Epi Info-7. We calculated frequencies and proportions for categorical variables and calculated means and medians for the continuous variables. We calculated prevalence odds ratios (PORs) and their 95% confidence intervals (CI); p-values ≤ 0.05 were considered statistically significant. Variables with a Chi-square test with p-value ≤ 0.15 were included in a logistic regression model using a backward stepwise elimination method to identify independently associated factors.

### Ethical approvals and considerations

We obtained written, informed consent from each participant before conducting the interviews. Permission to conduct the study in the selected health facilities was granted by the Kisumu County Health Authorities. Ethical clearance for this study was obtained from Jaramogi Oginga Odinga Teaching and Referral Hospital Ethical Review Committee (ERC.IB/VOL.I/130).

## Results

### Socio-demographic characteristics of respondents

We recruited a total of 216 female study participants. Their mean age was 26.1 years (standard deviation ± 5.5 years). One hundred and thirty one (61%) lived in urban Kisumu City. There were 189 (87.5%) women who were married, 20 (9.3%) were single, five (2.3%) were separated from their husbands and two (0.9%) were widowed. Twenty five of the married women (13.2%) were in polygamous relationships. The mean age at the time of marriage was 20.9 years (± 3.8 years). Those who had > 8 years of formal schooling were 119 (55.1%) and 32 women (14.8%) were formally employed. The median number of children that the women had was two (range 1-9 children).

Fifty (23.3%) women were HIV-positive, with 24 (48%) diagnosed during their ANC visit. There were 204 women (94.4%) who attended ANC in public health facilities; 75 (36.8%) of them had attended ANC less than four times during pregnancy. Male partner employment status during pregnancy varied: 65 (30.1%) were unemployed, 44 (20.4%) were formally employed, and 107 (49.5%) were self-employed (Table 1).

**Table 1:** Socio demographic characteristics of women attending child immunization clinic in Kisumu East Sub County, Western Kenya, 2015

Variable	N=216	Percent
**Health Centre**		
Lumumba (Winam Division)	106	49.1
Migosi (Winam Division)	24	11.1
Rabuor (Kadibo Division)	58	26.9
Nyangande (Kadibo Division)	28	12.9
**Age group (Years)**		
18-22	72	33.3
23-27	62	28.7
28-32	52	24.0
33-37	22	10.2
38-47	8	3.7
**Total No of children**		
1-2	130	60.2
3-5	78	36.1
> 5	8	3.7
**Marital Status**		
Married	189	87.5
Single	20	12.5
Separated	5	2.3
Widowed	2	0.9
**Education level**		
Primary	97	44.9
Secondary	75	34.7
Tertiary	44	20.4
**Religion**		
Christians	184	82.2
Traditional African Religion	26	12
Muslim	6	2.8
**Employment status**		
Unemployed	109	50.5
Self-employment	75	34.7
Formal employment	32	14.8
**Residence**		
Rural	85	39
Urban	131	61

### Level of male involvement

Forty-eight (22%) women had been accompanied to the ANC on at least one occasion by their partner and 31(14%) of the men were tested for HIV during ANC visit. Among the 216 respondents, there were 207 (96%) women who reported that they verbally informed their partners about the ANC appointments, 141 (66%) women who had discussed the ANC interventions with their partner. The women reported that67 (31%) of their partners had read the mother-child booklet during ANC. Among these women, 64 (96%) were married and 43 (64%) had been accompanied by the partner to the ANC. There were 174 (81%) women who had received financial support from their partner to attend ANC (Table 2). Therefore, forty-two women had high male involvement, representing 19% (95% CI 14-24%) of all women interviewed.

**Table 2:** Male involvement in elimination of mother to child transmission, Kisumu East sub County, Western Kenya, 2015

Component of male partner involvement index	Yes (%)	No (%)
The Woman was accompanied by the man to the ANC on at least one visit	48 (22)	168 (78)
The man was tested for HIV during any one of those visits	31(14)	185 (86)
The woman reported that the man was aware of the woman’s ANC appointments and discussed ANC interventions with the woman	141(65)	66 (45)
The man consulted the woman on the use of condoms and other contraception.	83 (60)	56 (40)
The man provided financial support for the woman’s ANC attendance on at least one occasion	174 (81)	42 (19)

### Factors associated with high male involvement

High male involvement was associated with women who had ≥ 8 years of formal schooling (POR 5.4, CI = 2.3–12.7) compared to women who had < 8 years formal schooling, having undergone couple HIV testing before pregnancy (POR 4.3, CI=2.0-9.2) compared to those who had not undergone couple testing before their pregnancy. It was also associated with the male partner being employed (POR 3.9, CI = 1.5-10.4) compared to when male partner was unemployed, the woman being employed (POR 3.7, CI = 1.6-8.2) compared to those women who were unemployed, and reports of the male partner having read the mother-child booklet during pregnancy (POR 3.2, CI = 1.6-6.3) compared to those who did not read the mother-child booklet during pregnancy. Those women who were aged > 21 years at the time they got married (POR 3.1, CI = 1.5-6.4) were also more likely to experience high male involvement compared to the women who were ≤ 21 years at the time of marriage. Conversely, women whose partner was aged < 24 years were less likely to experience high male involvement (POR 0.2, CI = 0.02-0.9) compared to those whose partner was aged > 24 years, and women who had attended public health facilities for ANC (POR 0.2, CI=0.1-0.7) were less likely to experience high male involvement compared to those who had attended ANC in private health facilities (Table 3).

**Table 3:** Factors associated with high level male involvement in Kisumu East sub County, Western Kenya, 2015

Variable	Male involvement		Unadjusted POR (95% CI)	Adjusted POR (95%CI)
	High	Low		
**Age in years**				
18-24	10 (12)	73 (88)	2.3 (1.1-5.0)	4.2 (0.4-45.6)
24	32 (14)	101 (76)	Reference	Reference
**Marital Status**				
Married	40 (21.2)	149 (78.8)	3.3 (0.8–14.8)	2.1 (0.4–10.9)
Others	2 (7.4)	25 (92.6)	Reference	Reference
Age at time of marriage				
>21 years	26 (31.7)	56 (68.3)	3.1 (1.5–6.4)	1.5 (0.6–4.0)
≤ 21 years	14 (13.1)	93 (86.9)	Reference	Reference
Level of Education				
>8 years of formal schooling	35 (29.4)	84 (70.6)	5.4 (2.3–12.7)	3.9 (1.5–10.1)
≤8 years of formal				
schooling	7(7.2)	90 (92.8)	Reference	Reference
Employment Status				
Employed	13 (40.6)	19 (59.4)	3.7 (1.6–8.2)	2.4 (0.9–6.7)
No employment	29 (15.8)	155 (84.2)	Reference	Reference
Residence				
Rural	31 (23.4)	100 (76.3)	2.1 (0.9–4.4)	1.3 (0.5–3.1)
Urban	11 (12.9)	74 (87.1)	Reference	Reference
**Partner employment status**				
Employed	37 (24.5)	114 (75.6)	3.9 (1.5–10.4)	3.3 (1.2–9.5)
Unemployed	5 (7.7)	60 (92.3)	Reference	Reference
Male partner read mother child booklet				
Yes	22 (32.8)	45 (67.2)	3.2 (1.6–6.3)	2.9 (1.3–6.5)
No	20 (13.4)	129 (86.6)	Reference	Reference
History of couple testing before pregnancy				
Yes	31 (31.3)	68 (68.7)	4.3 (2.0–9.2)	3.2 (1.4–7.2)
No	11 (9.6)	104 (90.4)	Reference	Reference
Woman attended public facility for ANC				
Yes	36 (17.6)	168 (82.4)	0.2 (0.1–0.7)	0.4 (0.1–1.6)
No	6 (50.0)	6 (50)	Reference	Reference

+CI-Confidence Interval; +POR-Prevalence Odds Ratio

Adjusting for factors simultaneously, we found that women who had > 8 years of formal schooling (AOR 3.9, CI = 1.5-10.1), history of prior couple testing (AOR 3.2, CI = 1.4-7.2), instances where the male partner was employed (AOR 3.3, CI = 1.2-9.5) and the partner having read the mother-child booklet after woman’s ANC visit (AOR 2.9, CI = 1.3-6.5) remained associated with high male involvement (Table 3).

## Discussion

From our findings, high male involvement was uncommon, as reported by one out of every five women. Four factors: woman’s level of education, male partner employment, male partner reading the mother-child booklet during ANC period and history of prior couple testing before pregnancy were associated with high male involvement.

The proportion of women with high male involvement in our study was lower than findings in Uganda, where 26% of the women experienced high male involvement [16]. However, our study included male partner testing for HIV at the ANC as one of the variables in the index, which was not assessed by the study in Uganda. In our study the proportion of women reporting male partner testing for HIV at the ANC (14.4%) was comparable several other Kenyan studies that defined male involvement as male partner HIV testing at the ANC and found 10%-16.5% involvement [6,20,21]. Our study area has a high HIV prevalence of 15.1% [7] and there has been significant improvement in the programs geared towards eMTCT in the area, but the low prevalence of high male involvement implies the need to sensitize men of the importance of their involvement in eMTCT activities.

Similar to other studies in Sub Saharan Africa [5,12,17,26], we identified a positive association between high male involvement and women with at least 8 years of education. In another study in Kenya, women who had attained at least 9 years of formal education were more likely to experience male involvement [27].Woman’s level of education might influence partner involvement because education can lead to improved access to information, and because more educated women could be more able to discuss matters affecting their reproductive health with their partners [12].

Women in our study with employed male partners were more likely to report high male involvement, consistent with studies in Rwanda and Malawi [28,29]. In Uganda, a study that used a male involvement index found that women whose partners were not formally employed were less likely to experience high male involvement compared to those whose partners were formally employed [16]. It is plausible that unemployed men might be more likely to go in search of a daily income, and so they might view going to the ANC as a missed opportunity for finding work. This implies the need for a system that would encourage these men to visit the ANC with their partners that does not interfere with their efforts to participate in daily work or allows them to earn wages in or around the setting of the ANC while their partner visits.

A history of prior couple testing was also associated with high male involvement, a finding similar to those in Eastern Uganda [16]. One explanation for this could be that these men were already aware of their HIV-infection status, and therefore less apprehensive of the HIV test outcome. Another study in Ethiopia, which based male involvement on male partner ANC attendance reported a similar finding [15]. We believe that prior couple testing improves the man’s knowledge of HIV and their role in eMTCT, as has been shown elsewhere [26,30-32]. Couple testing has also been shown to have other benefits including reducing the rates of adverse events that may discourage women from attending ANC like domestic violence [20,31].

Women who reported that their partners had read the mother-child booklet during ANC were also more likely to experience high male involvement. It is possible that these men had a better understanding of ANC services compared to other men who were verbally informed but never read the booklet, or it might be an indicator for the partners’ general interest or attention to mother-child health. In our study area, women generally inform the men verbally on the need to visit the ANC or to participate in eMTCT activities. Only 22% of the women in our study were accompanied by the male partner to the ANC despite 96% of them reporting that they verbally informed the male partner on the need to visit the ANC. Formal invitation using a card was reported to improve male involvement in past studies [16,33,34] and could improve male involvement by up to 10% [35], while verbally inviting them has been shown to have little improvement on male involvement [16,27]. Our study area might benefit from a formal partner notification system to improve the level of male involvement, for which various methods such as formal invitation via card or SMS could be investigated.

This study is not without limitations. There was potential bias by interviewing only the women, as they might have misreported information about their partners. Future studies ideally should include interviews directly with male partners, and could also validate answers between men and women. Mothers may potentially have poor recall for their partners’ involvement. We minimized the potential of recall bias by including only women with children aged ≤ 12 months and by referring to the mother-child booklet to verify mother’s socio demographic information. We used an index used in similar studies, and we do not know the reliability of this index, however, the components used in the index seemed rationale to our study setting. The index we used may or may not be generalizable to other settings, and we do not know the validity or reliability of the index, however, many of our findings was consistent with other studies of male partner involvement. As a cross-sectional study, our factors identified may not necessarily represent causal factors as may better be identified through a cohort study following partners over time, however, the factors we identified are consistent with other studies on male partner involvement. We also did not study attitudes and beliefs of women or their partners, which may influence behavior and subsequently influence male partner involvement. Finally, this study’s size was designed based on estimating a proportion of women with male partner involvement, therefore our risk factor analysis was a secondary aim, and the study not specifically powered to detect risk factors with high precision.

## Conclusion

We found that few women reported a high level of male involvement in a region of high HIV prevalence. Male partner involvement in our study area was associated with woman’s level of education, the partner’s employment status, and the male partner reportedly reading the mother-child booklet. Our study findings imply need for a change in policy considerations that improve male involvement such as those that target women with lower levels of education, those with unemployed partners, or those which review methods for joint review of the mother-child book between partners. Other ideas such as introducing a method of invitation of men to visit the ANC could be investigated by the Kisumu County health authorities and general awareness campaigns on the need for men to participate in eMTCT activities.

### What is known about this topic

- Male partner involvement in activities targeting eMTCT is a key strategy that can significantly lower rates of MTCT;
- Studies have been undertaken in various countries on the factors related to male partner involvement, and these studies reviewing male partner involvement have used various indicators, some of which combine indicators to create a male partner involvement index.

### What this study adds

- We found that despite the high prevalence of HIV in Kisumu East sub County in Western Kenya, the area has low levels of male involvement in eMTCT activities;
- This study identifies the modifiable factors that influence male involvement in eMTCT and hence is useful in developing targeted interventions to improve male involvement.

## Competing interests

The authors declare no competing interest.
